# Experimental data of deformation and cracking behaviour of concrete ties reinforced with multiple bars

**DOI:** 10.1016/j.dib.2017.05.038

**Published:** 2017-05-25

**Authors:** Arvydas Rimkus, Viktor Gribniak

**Affiliations:** Research Laboratory of Innovative Building Structures, Vilnius Gediminas Technical University, Vilnius, Lithuania

**Keywords:** Reinforced concrete, Multiple bars, Tensile tests, Deformations, Cracking

## Abstract

The data presented in this article are related to the research article entitled “Experimental Investigation of cracking and deformations of concrete ties reinforced with multiple bars” (Rimkus and Gribniak, 2017) [1]. The article provides data on deformation and cracking behaviour of 22 concrete ties reinforced with multiple bars. The number and diameter of the steel bars vary from 4 to 16 and from 5 mm to 14 mm, respectively. Two different covers (30 mm and 50 mm) are considered as well. The test recordings include average stains of the reinforcement and the concrete surface, the mean and maximum crack spacing, final crack patterns, and crack development schemes obtained using digital image correlation (DIC) system. The reported original data set is made publicity available for ensuring critical or extended analyses.

## **Specifications Table**

**Table:** 

Subject area	*Civil Engineering*
More specific subject area	*Construction Materials; Structural Engineering*
Type of data	*Tables, Figures, Excel datasheet*
How data was acquired	*The reaction of the ties was measured with the electronic load cells of the testing machines; the axial deformations of the ties were monitored using linear variable displacement transducers (LVDT); surface deformation and crack development were obtained by using the digital image correlation system* with the aid of *DaVis 8.1.6* software by *LaVision.*
Data format	*Raw, Filtered and processed*
Experimental factors	*During the curing process, the ties were stored in water to reduce the shrinkage effect. The specimens were tested at the laboratory conditions.*
Experimental features	*Special testing equipment has been developed to test concrete ties reinforced with multiple bars. The tensile tests were carried out using electromechanical and servohydraulic machines under displacement control with 0.2 mm/min loading.*
Data source location	*Vilnius, Lithuania*
Data accessibility	*Data within this article.*
Related research article	*A. Rimkus, V. Gribniak, Experimental Investigation of cracking and deformations of concrete ties reinforced with multiple bars, Construction and Building Materials 148 (2017), pp. 49–61. DOI:*10.1016/j.conbuildmat.2017.05.029.

## **Value of the data**

• The test data allows investigating effect of distribution of bar reinforcement on deformation and cracking behaviour of concrete elements subjected to tension.
• The data allows assessing effect of variation of the diameter-to-reinforcement ratio on cracking behaviour of concrete ties.
• The reported data makes a base for developing a material model of the tensile concrete.

## 1 Data

**Fig. 1:**
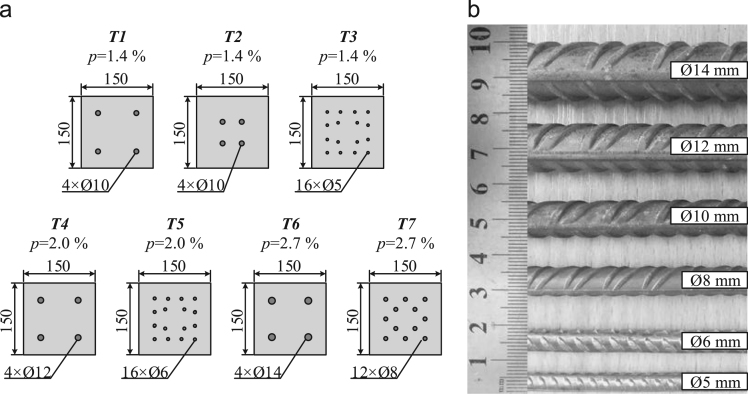
Tested elements (all dimensions in mm): cross-sections of the ties (a), reinforcement bars (b).

**Fig. 2:**
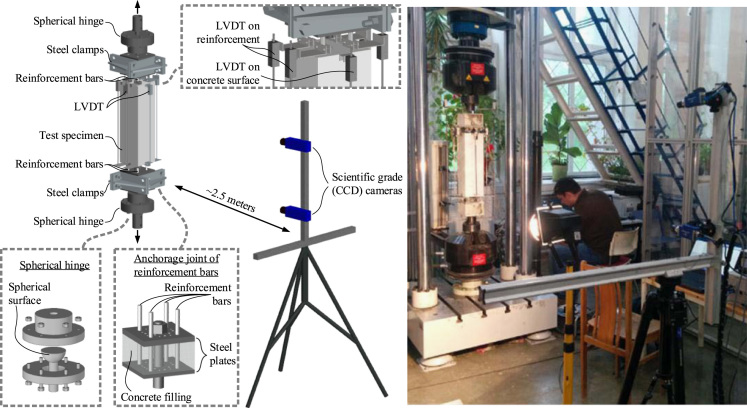
Test setup of the tie tests.

The experimental recordings reported in this paper were collected during the tests of concrete ties reinforced with multiple bars [1]. Cross-sections of the ties and surface characteristics of the reinforcement bars are presented in Fig. 1 with main reinforcement characteristics described in Table 1. The tests were performed in the Research Laboratory of Innovative Building Structures at Vilnius Gediminas Technical University (Lithuania). The test setup is shown in Fig. 2. Crack spacing characteristics and crack development schemes are presented in Table 2, Table 3, respectively. The Supplementary material consists of the database of average strains of reinforcement and concrete surface of the ties.

**Table 1:** Main characteristics of the reinforcement.

Type	Reinforcement, mm	*c*, mm	*p*, %	∅/*p*, mm	*f*_*y*_, MPa	*E*_*s*_, GPa
*T1*	4×∅10	30	1.4	714	510.1	199.5
*T2*	4×∅10	50	1.4	714	510.1	199.5
*T3*	16×∅5	30	1.4	357	503.9	200.7
*T4*	4×∅12	30	2.0	600	543.7	202.8
*T5*	16×∅6	30	2.0	300	504.7	203.6
*T6*	4×∅14	30	2.7	519	558.7	205.3
*T7*	12×∅8	30	2.7	296	473.9	197.1

**Table 2:** Crack spacing of the ties (parameters of the ties *P1-4*×*10*, *P1-16*×*5*, and *P2-12*×*8* were not identified).

Type	Name	*f׳*_*c*_, MPa	*f*_*cm*_, MPa	Age, days	Length of the prism, mm	*s*_*max*_, mm	*s*_*m*_, mm	*s*_*max*_*/s*_*m*_
*T1*	*P1-4*×*10*	39.5	38.6	34	379	–	–	–
	*P2-4*×*10*	41.3	39.5	41	383	184	127	1.45
	*P3-4*×*10*	43.1	46.7	16	503	117	83	1.40
	*P4-4*×*10*	43.1	46.7	16	504	125	100	1.25
	*P5-4*×*10*	40.8	44.7	15	504	134	100	1.34
	*P6-4*×*10*	40.8	44.7	15	496	196	167	1.18
*T2*	*P7C-4*×*10*	40.8	45.3	14	496	235	167	1.41
	*P8C-4*×*10*	40.8	45.3	14	498	207	167	1.24
*T3*	*P1-16*×*5*	39.5	38.6	34	381	–	–	–
	*P2-16*×*5*	41.3	39.5	41	388	118	100	1.18
	*P3-16*×*5*	43.1	46.7	16	503	115	83	1.38
	*P4-16*×*5*	43.1	46.7	16	500	90	63	1.44
*T4*	*P1-4*×*12*	59.6	54.0	77	503	130	71	1.82
	*P2-4*×*12*	59.6	54.0	77	505	165	100	1.65
*T5*	*P1-16*×*6*	59.6	54.0	77	493	135	83	1.62
	*P2-16*×*6*	59.6	54.0	77	496	130	100	1.30
*T6*	*P1-4*×*14*	43.6	43.6	28	494	127	100	1.27
	*P2-4*×*14*	44.0	43.5	31	498	129	100	1.29
	*P3-4*×*14*	44.2	43.2	34	497	125	100	1.25
*T7*	*P1-12*×*8*	43.6	43.6	28	489	170	100	1.70
	*P2-12*×*8*	44.2	43.2	34	494	–	–	–
	*P3-12*×*8*	44.2	43.2	34	499	122	100	1.22

**Table 3:** Final crack pattern and crack development schemes of differentt types (“DNE”=does not exist).

## 2 Design of the tests, materials, and methods

### 2.1 Test specimens

The test campaign consists of 22 ties with different arrangement of the reinforcement. Seven types of the sections (denoted to as *T1*–*T7*) are shown in Fig. 1. Each of the tie groups with the same reinforcement ratio *p* consists of two or three different reinforcement schemes: 4-bars reference and 16-bars (or 12-bars) alternative. Two different covers (i.e. 30 mm and 50 mm) are considered in the ties with the lowest ratio *p* (Fig. 1a). Six different bar diameters (Fig. 1b) are used. The main characteristics of the reinforcement are presented in Table 1, where the first two columns refer to the type of the tie and the reinforcement arrangement scheme (including the number and diameter of the bars). Other parameters presented in the table are the cover (*c*), the reinforcement ratio (*p*), the ratio of the bar diameter to the reinforcement ratio (∅/*p*), the yield strength (*f*_*y*_) and modulus of elasticity (*E*_*s*_) of the bars.

Gribniak and Rimkus [2] have developed specific equipment for anchorage of multiple bars as shown in Fig. 2. The anchorage joints embrace two plates connected by a central bar that is connected to the tension device using a spherical hinge. The latter allows reducing a possible imperfection in applying the tensile load. The plates are perforated to fix and distribute the reinforcement bars within the concrete prism. Steel clamps are used for ensuring the confinement of the anchorage joints.

Specimens were produced in six batches using similar concrete mixture with a maximum aggregate size of 8 mm and the target compressive strength class of C30/37. The modulus of elasticity and compressive strength of the concrete were determined using ∅150×300 mm cylinders. All test samples were stored in water to reduce the shrinkage effect. Characteristics of the concrete are given in Table 2, where the first two columns refer to the type and name of the tie. The table includes the average compressive strength of the concrete on the testing day (*f׳*_*c*_) and at 28 days (*f*_*cm*_) as well as the age of the specimen on the testing and the length of the concrete prism.

### 2.2 Tie tests

Initially, the tests were performed using an electromechanical machine of 100 kN capacity. However, due to the limitation of the length of the concrete prism (≈380 mm), the main tests were carried out using a servohydraulic machine of 600 kN capacity under displacement control with 0.2 mm/min loading. The reaction was measured with the electronic load cells of the testing machines. The axial deformations were monitored using linear variable displacement transducers (LVDT), which were attached to the reinforcement bars and to the concrete surface as shown in Fig. 2. Average strains of the reinforcement and concrete surface assessed by using the LVDT recordings are reported in Appendix A.

In order to observe the development of the cracks, the front surface of the ties was exposed to a digital image correlation (DIC) system. Images were captured by two digital cameras (Imager E-lite 5M) placed on a tripod at 2.5 m distance from the test specimens. The cameras, incorporating a charge-coupled device (CCD) detector, have a resolution of 2456×2085 pixel at 12.2 fps frame rate.

The final crack patterns of the ties are presented in Table 3. This table also shows development of the cracks (at the surface denoted to as “DIC”) identified, following the methodology described in [3], by the DIC system with *DaVis 8.1.6* software by *LaVision*. The cracking schemes are related to the reference average strains of the reinforcement (*ε*_*s*_). The maximum (*s*_*max*_) and average crack distances (*s*_*m*_) of the ties determined at stabilized cracking stage are given in Table 2. The stabilized cracking stage is associated with the average strain *ε*_*s*_≈1.5‰ and 1.0‰ for the ties with *p*≤2.0% and *p*=2.7%, respectively.

## Funding sources

This work is part of a Ph.D. thesis of Arvydas Rimkus and was funded by the Research Council of Lithuania (Research Project MIP–050/2014).

## Footnotes

Appendix A Supplementary data associated with this article can be found in the online version at http://dx.doi.org/10.1016/j.dib.2017.05.038.
